# Liquid-liquid triboelectric nanogenerator based on the immiscible interface of an aqueous two-phase system

**DOI:** 10.1038/s41467-022-33086-2

**Published:** 2022-09-09

**Authors:** Ye Lu, Longlong Jiang, Yang Yu, Dehua Wang, Wentao Sun, Yang Liu, Jing Yu, Jun Zhang, Kai Wang, Han Hu, Xiao Wang, Qingming Ma, Xiaoxiong Wang

**Affiliations:** 1 College of Physics, Qingdao University, 266071 Qingdao, China; 2 School of Pharmacy, Qingdao University, 266071 Qingdao, China; 3 University-Industry Joint Center for Ocean Observation and Broadband Communication, College of Physics, Qingdao University, 266071 Qingdao, China; 4 Weihai Innovation Research Institute of Qingdao University, Weihai, 264200 PR China; 5 Collaborative Innovation Center for Eco-Textiles of Shandong Province, and State Key Laboratory of Bio-Fibers and Eco-Textiles, Qingdao University, 266071 Qingdao, China; 6 School of Health and Life Sciences, University of Health and Rehabilitation Sciences, 266113 Qingdao, China; 7 Microsystem and Terahertz Research Center, China Academy of Engineering Physics, 610200 Chengdu, China; 8 Institute of Electronic Engineering, China Academy of Engineering Physics, 621999 Mianyang, China; 9 School of Physics and Electronics, Shandong Normal University, 250014 Jinan, China; 10 School of Electrical Engineering, Qingdao University, 266000 Qingdao, China; 11 State Key Laboratory of Heavy Oil Processing, College of Chemical Engineering, China University of Petroleum (East China), 266580 Qingdao, China; 12 School of Mathematics and Statistics, Qingdao University, 266071 Qingdao, China

**Keywords:** Materials for energy and catalysis, Devices for energy harvesting, Fluids, Electron transfer, Fluid dynamics

## Abstract

Solid nanogenerators often have limited charge transfer due to their low contact area. Liquid–liquid nanogenerators can transfer a charge better than the solid–solid and solid–liquid counterparts. However, the precise manipulation of the liquid morphology remains a challenge because of the fluidity limits of the liquid. In this work, using the surface tension of a droplet to fix its shape, a liquid-liquid triboelectric nanogenerator in Contact-Separation mode is designed using an immiscible aqueous-aqueous interface, achieving a contact surface charge transfer of 129 nC for a single droplet. The configuration is proven to be applicable in humid environments, and the two-phase materials have good biocompatibility and can be used as an effective drug carrier. Therefore, this nanogenerator is useful for designing future implantable devices. Meanwhile, this design also establishes the foundation of aqueous electronics, and additional applications can be achieved using this route.

## Abstract (web summary)

While liquid-liquid interface offers better contact and charge transfer potential than solid-based counterparts, fluidity still poses challenges for their application. Here, authors show that charge transfer exists in aqueous two-phase systems and propose a nanogenerator design based on the immiscible aqueous-aqueous interface.

## Introduction

A nanogenerator (NG) is a device for utilizing distributed energy^1–4^. Based on their energy-conversion principles, nanogenerators can be divided into various categories, including piezoelectric nanogenerators (PENGs)^5–9^, triboelectric nanogenerators (TENGs)^10–15^, and pyroelectric nanogenerators (PyENGs)^16–19^. Among these, TENGs have received significant scientific interest because of their unique characteristics, which include greater output energy^20^. The contact separation mode of a TENG provides an important repeatable and quantitative research scheme. In general, the basic working principle of a TENG relies on the displacement current induced by the charge, whose energy output depends on the enlargement of the microscopic contact area of the interface and subsequent increase in the interface electrons in the cloud overlap, which generate a larger charge transfer^21–24^. Traditional TENGs are typically designed based on a solid–solid interface^10,25–27^. Recently, following the creative design of ref. 28, nanogenerators based on solid–liquid interfaces have attracted increasing attention^28–30^. However, it is difficult to achieve 100% contact in either a solid–solid or solid–liquid interface, prohibiting the practical applications of TENGs. In contrast, 100% contact can easily be achieved in a relatively straightforward manner using a liquid–liquid interface, which shows great promise for developing TENGs with effective contact areas^31–35^. A liquid–liquid TENG (L-L TENG) was first proposed by Chen et al. in 2019^36^, extending TENGs from solid–solid and solid–liquid friction to liquid–liquid friction, and representing an important paradigm shift for future scientific research on TENGs. The first proposed L-L TENG was irreversible^36,37^. Then, in 2020, Wang et al. designed a reversible electrostatically induced L-L TENG using magnetofluidics^38^. However, the output efficiencies of the current L-L TENGs are relatively low, and L-L TENGs that can realize usable energy outputs are urgently needed. The difficulty can be attributed to the lack of suitable materials for designing L-L TENGs, mainly related to the fusion and dissolution of the two liquids, which cannot be effectively separated after contact electrification. Therefore, finding materials that can be efficiently separated after contact electrification is the key issue for developing a high-performance L-L TENG. L-L TENG designs can reference the modes of the original solid–solid nanogenerators, which include the vertical contact-separation mode, lateral sliding mode, single-electrode mode, freestanding mode, and electrostatic induction mode^8,15,38–42^. The commonality of these modes is the periodic change in the interface capacitance. Therefore, the construction of a periodic change in capacitance at the liquid–liquid interface is the basis for developing more reliable L-L TENGs.

Traditional liquid–liquid interfaces have relatively high viscosities, and it is difficult to achieve effective contact separation between the liquids. In recent years, new liquid–liquid phase separation control technology based on all-aqueous systems has been developed that can be effectively used for reference^43^. In particular, when two water-soluble polymers, or one water-soluble polymer and one salt, are dissolved and mixed at an appropriate concentration and temperature, if the reduction in enthalpy in the system is sufficient to overcome the energy cost associated with the increase in entropy, these different solutes can redistribute in the water and trigger phase separation, forming an aqueous two-phase system (ATPS), by which a recoverable immiscible aqueous–aqueous interface (IAAI) can be created^44–46^. Compared with a traditional water–oil interface, the viscosity and interfacial tension of the IAAI are lower, which can achieve more effective contact separation^46,47^. Such a recoverable interface provides a platform for deeper research on material charge transfer, which will benefit future research in the NG fields. In addition, the ATPS can avoid the use of oily phases, organic solvents, strong acids, and alkalis with poor biocompatibility. Thus, it can provide a green all-aqueous environment and endow superior biocompatibility for the entire research process, which enables the construction of biomimetic nanogenerators.

In this work, an IAAI control case was demonstrated that made it possible to develop L-L TENGs. Such L-L TENGs in series could supply power to an external circuit. The construction of the IAAI and the design of controlled electronic devices provide important examples for the design of liquid-phase electronic devices in the future. Based on this interface, more detailed research could be conducted to gain a better understanding of the charge behavior at the liquid–liquid interface.

## Results and discussion

### Material characterization and solution differences

**Fig. 1:**
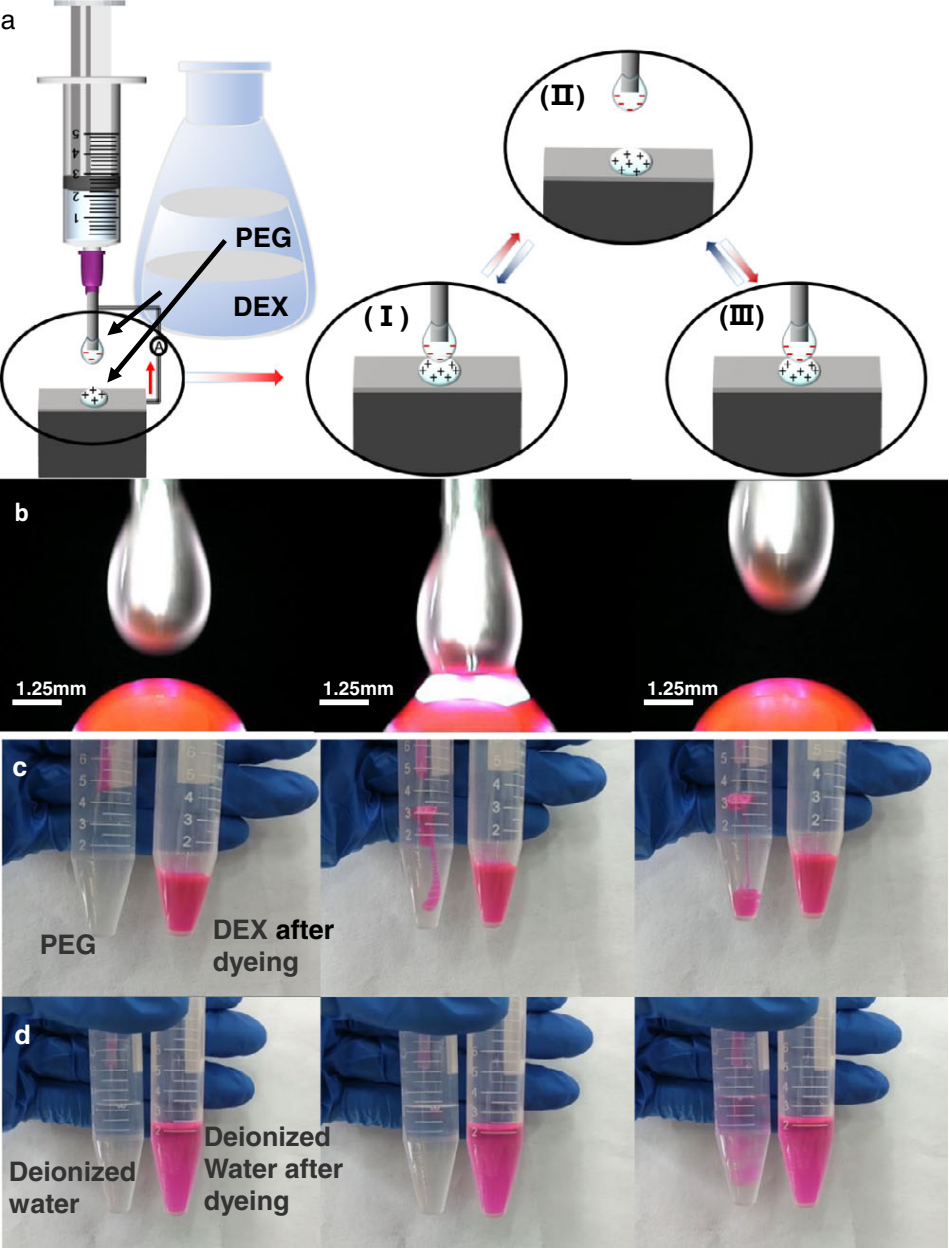
Demonstration of the L-L TENG based on the aqueous two-phase system. **a** Contact-separation working mode of the L-L TENG. **b** Optical images of DEX droplet and dyed PEG droplet coming into contact and separating. **c** Images of dyed DEX droplet dripped into PEG to form an IAAI. **d** Optical images of a dyed deionized water droplet dripped into undyed deionized water without the formation of an IAAI.

As shown in Fig. 1a, the upper droplet was suspended from the tip of a needle, which simultaneously acted as an electrode. A piece of aluminum foil acted as the other electrode and supported the lower droplet. The syringe was driven by a stepping motor that moved the droplets up and down periodically to achieve the contact and separation of the two droplets. In the initial state (I), the PEG droplet was in contact with the DEX droplet, and there was no current or potential. As a result of the surface electron affinity difference, a charge (electron or ion) was transferred from the surface of the PEG droplet to the surface of the DEX droplet, leaving a net positive charge on the surface of the PEG droplet and net negative charge on the surface of the DEX. When separating, in a case where electrons can flow through an external circuit (state (II)), the resulting charge separation will induce a potential difference between the electrodes, causing the subsequent current flow. At the maximum separation distance, the positive current was reduced to zero (state (III)). When the two charged surfaces again came into contact, the current reversed^48–50^. Without any contact, no charge was transferred.

In order to characterize the droplet morphology and compatibility of the L-L TENG during its operation, an optical microscope was used to monitor the three periods before, during, and after the contact separation, as shown in Fig. 1b. It can clearly be seen that there was little change in the size and morphology of the droplets before and after the contact and separation. In addition, the color of the DEX droplets did not change (Supplementary Movie 1). This demonstrated that the droplets did not dissolve into each other during the contact and separation. In order to further verify the compatibility of the PEG and DEX solutions, DEX dyed with rhodamine B dye was added dropwise to the PEG solution, as shown in Fig. 1c, and compared with dyed deionized water droplets added to pure water, as shown in Fig. 1d. When the dyed deionized water droplets entered the water, they rapidly dissolved to form a single phase, and the color diffused. In contrast, there was no color diffusion phenomenon when the DEX droplet entered the PEG solution. Because of the density difference, the DEX droplet slowly settled to the bottom of the test tube, and obvious delamination occurred, resulting in a stable IAAI (Supplementary Movie 2). This provided sufficient support for the PEG and DEX contact and separation movement without dissolving into each other.

**Fig. 2:**
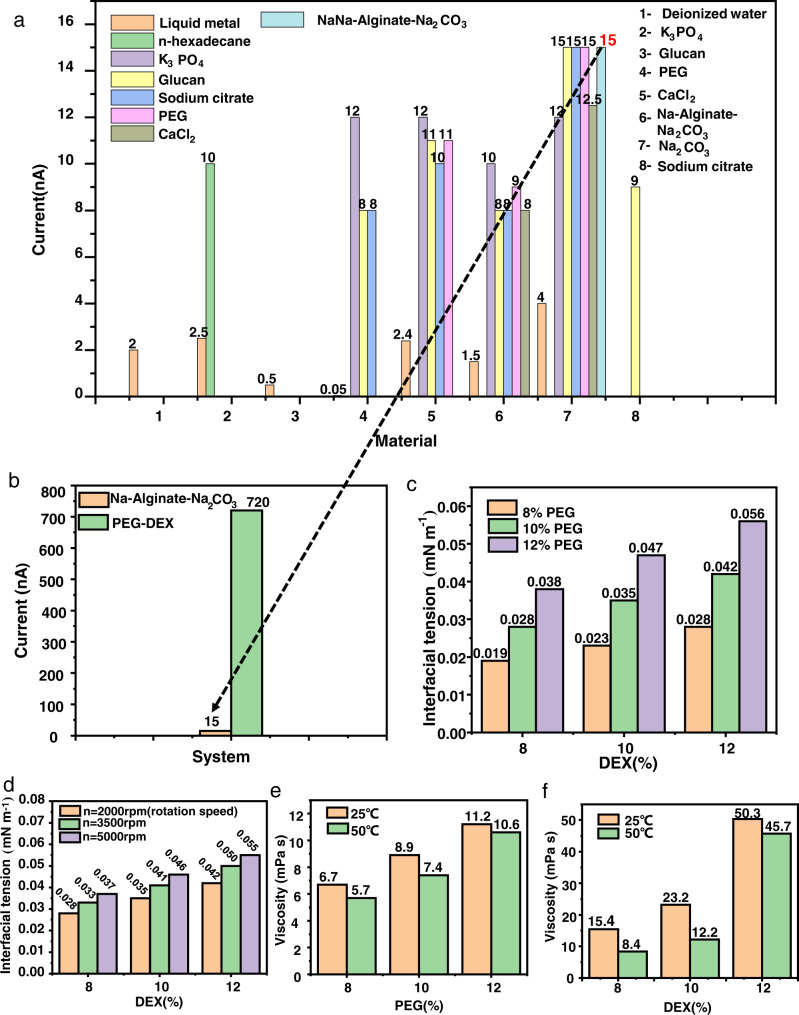
Miscibility and output between various aqueous two-phase systems. **a** Output signal from contact separation of multiple liquid systems. **b** The output contrast between the PEG-DEX and NaA–Na_2_CO_3_ systems. **c** Surface tension variation with the liquid concentration. **d** Effect of rotating speed on the surface tension of DEX when the PEG concentration was 10%. **e** Variation of PEG viscosity with temperature. **f** Variation of DEX viscosity with temperature.

The contact-separation electrification performances of a series of liquids were tested to determine the best one. Although some liquids would dissolve or react when contacted (as listed in Supplementary Table 1), there was still a current signal. Figure 2a shows the closed-circuit current signal generated by the contact and separation of different selected liquids under the same condition. It can clearly be seen that the maximum closed-circuit current signal of the other liquid pairs was 15 nA. However, the output signal of the PEG-DEX pair prepared by ATPS was ~48 times that of the previous combination, as shown in Fig. 2b. It was speculated that the larger amount of charge transfer was related to the immiscibility of the two droplets. In order to verify this speculation, we measured the closed-circuit currents for a group of liquid–liquid contact immiscible (PEG-DEX), liquid–liquid contact miscible (sodium citrate–Na_2_CO_3_), and liquid–liquid contact reactive (CaCl_2_–Na_2_CO_3_) cases, as shown in Supplementary Fig. 4. It could easily be seen that the current output of the immiscible liquid–liquid interface contact separation was significantly larger than that of the miscible case. Moreover, the current output in the miscible case only existed for the very short time of the contact, while in the case of the immiscible contact separation, sufficient current emerged as a result of the variation of the surface capacitance. According to $Q=\int {Idt}$, we could easily get the result that the liquid–liquid immiscibility had a significant effect on the enhancement of the charge transfer amount.

The L-L TENG droplets were characterized to better understand the system. The surface tension and viscosity of PEG and DEX were tested using the spinning drop method and rolling ball method, respectively. In the two-phase PEG-DEX system, when the concentration of DEX was constant, the surface tension of the PEG increased with its concentration. When the concentration of PEG was constant, the surface tension of the DEX increased with its concentration, as shown in Fig. 2c. At the same time, the surface tension measured by the spinning drop method could be affected by the rotational speed. Therefore, when the PEG concentration was 10%, the surface tensions of different proportions of DEX at different speeds were tested. As shown in Fig. 2d, when the concentration of DEX was constant, its surface tension gradually increased with the rotational speed. When the rotational speed was constant, the surface tension of DEX increased with its concentration. In summary, it can be seen that the surface tensions of PEG and DEX were mainly affected by their concentrations. In order to further characterize the PEG and DEX in this system, their viscosities were tested, and the results are shown in Fig. 2e, f, respectively. At the same temperature, the viscosity of PEG (DEX) increased with its concentration; at the same time, when the concentration of PEG (DEX) was constant, increasing the temperature decreased its viscosity. Because the DEX droplet was suspended from a needle, it did not easily fall off, and the viscosity had to be large. At the same time, in order to maintain the integrity of the interface during the droplet contact and separation, the surface tension of the droplet had to be as large as possible. Therefore, a PEG-DEX (10%–10%) system with moderate surface tension and viscosity was selected for further research. Unless otherwise specified, the L-L TENG tests were all conducted using this PEG-DEX (10%–10%) system at room temperature. Infrared spectroscopy was used to characterize the surface chemical bonds of the PEG and DEX powders, solutions, and products after the solutions were dried, and the influence of dissolution was analyzed. As shown in Supplementary Fig. 3a, before the dissolution step, the characteristic absorption bands of PEG were observed at 2876 cm^−1^, 1465 cm^−1^, and 1113 cm^−1^, which corresponded to the –CH_2_– symmetrical contraction vibration, –CH_2_– bending vibration, and COC flexural vibration, respectively. After the PEG dissolved, the absorption bands at 3419 cm^−1^, 1645 cm^−1^, and 1113 cm^−1^ could be attributed to the –OH stretching vibration, -OH bending vibration, and COC bending vibration, respectively. The main difference was that the intensity of the –OH peak had significantly increased. This showed that PEG had dissolved in deionized water. In the dried product, peaks corresponding to the –CH_2_– symmetrical contraction vibration, –CH_2_– bending vibration, and C–O–C bending vibration appeared again, indicating that the PEG was not destroyed. As shown in Supplementary Fig. 3b, before the dissolution step, the characteristic absorption bands of DEX were observed at 3419 cm^−1^ and 1013 cm^−1^, which corresponded to the –OH stretching vibration absorption peak and variable-angle vibration absorption peak of alcoholic hydroxyl groups, respectively. After dissolving the DEX, the absorption bands at 3419 cm^−1^, 1645 cm^−1^, and 1013 cm^−1^ could be attributed to the –OH stretching vibration, –OH bending vibration, and variable-angle absorption vibration peaks of alcoholic hydroxyl groups, respectively. The main difference was the –OH peak. The strength of the PEG peaks had significantly increased, indicating that the PEG was dissolved in deionized water. In the dried product, the –OH stretching vibration absorption peak and variable-angle vibration absorption peak of the alcohol hydroxyl group appeared, indicating that the DEX was not destroyed.

**Fig. 3:**
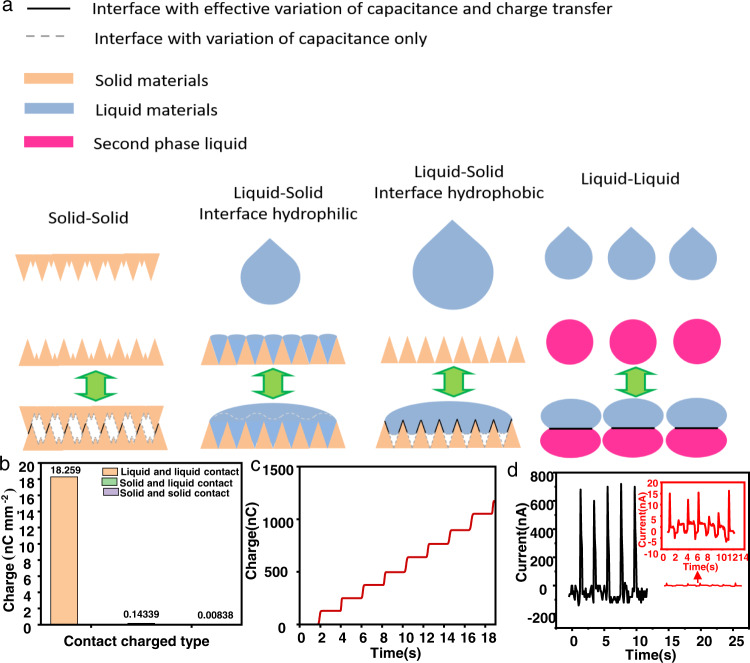
Interface comparison of liquid–liquid interface, liquid–solid interface, and solid–solid interface. **a** Solid–solid, solid–liquid, and liquid–liquid interface contact-separation mechanism analysis results. **b** Comparison of surface charge densities of solid–solid, solid–liquid, and liquid–liquid contacts. **c** Transferred charge densities of different matter states. **d** Closed-circuit current comparison of PEG-DEX in liquid–liquid and solid–solid interface contact separation.

The basic working principle of the triboelectric nanogenerator can be summarized as contact, charge transfer, capacitance change, and displacement current induction^51^. The common three states of matter are the solid, liquid, and gaseous states. Because the capacitance of gas is difficult to manipulate, there are relatively few studies on this type of material in the field of nanogenerators. Therefore, common triboelectric materials include solid–solid, solid–liquid, and liquid–liquid. Among these, in solid–solid contacts, only a small fraction of the interface area is close enough to achieve a charge transfer^22^. The following change in the relative positions of the two surfaces causes a change in capacitance, inducing a displacement current. Pressure may enlarge the working surfaces, but the overall change is small^52–54^. Solid–liquid interfaces could be divided into hydrophilic and hydrophobic situations. Although a hydrophilic interface can undergo a full charge transfer, the subsequent capacitance change interface does not coincide with the charge transfer interface, which makes it difficult to induce an effective displacement current. For a hydrophobic interface, if the interface is flat enough, the liquid can fully contact the solid, and it can produce a large output. However, if the common solid interface is not flat enough, the liquid cannot completely form a full interface with the solid, which causes the charge transfer interface to be unsatisfactory^55,56^. For an L-L TENG, if the liquid can be effectively separated, the charge transfer can be effectively realized on the droplet interface, and a full interface for charge transfer can be achieved, allowing an effective capacitance variation to be achieved. Such a contact benefit can be conveniently utilized in the future for the design of other electronic devices such as recoverable p-n junctions. In order to easily determine the charge transfer effects on different interfaces, a simple comparison experiment was developed, in which nylon-PVDF was used for the solid–solid contact, water droplet-PTFE was used for the solid–liquid contact, and the PEG-DEX aqueous solution pair was used for the liquid–liquid contact. As can be seen from Fig. 3b, for the corresponding charge transfer amount, the liquid–liquid contact achieved values on the ×10 nC/mm^2^ level, and the liquid–solid contact achieved values on the ×0.1 nC/mm^2^ level. The solid–solid contact achieved values on the pC/mm^2^ level, which was consistent with the results of the contact interface analysis. Figure 3c shows the repeated charge transfer steps of the L-L TENG. At the same time, we compared the closed-circuit currents of the PEG-DEX liquid–liquid interface and solid–solid interface contact separation (the solid–solid interface area is approximately four times the liquid–liquid interface area), as shown in Fig. 3d. It can clearly be seen that the closed-circuit current of the liquid–liquid interface contact was much larger than that of the solid–solid interface contact. The current in the liquid–liquid case was ~700 nA, while in the case of the solid–solid contact it was only ~15 nA. This further supported the previously stated point.

### Electrical performance analysis and simulation

**Fig. 4:**
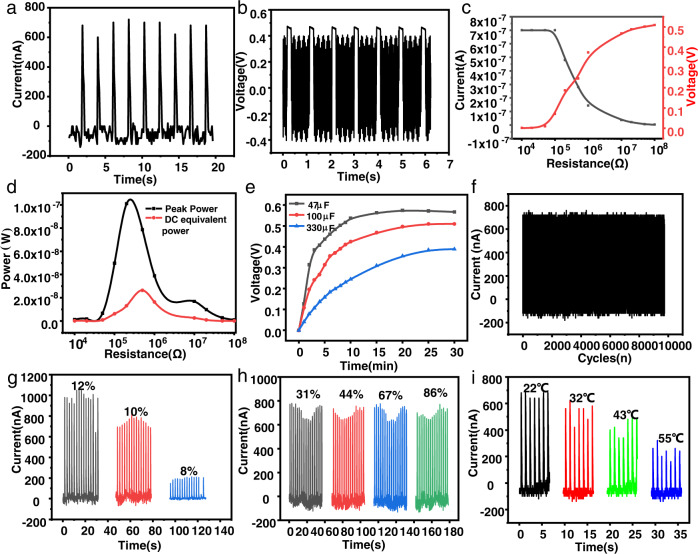
Output performance of the L-L TENG. **a** Closed-circuit current of the L-L TENG. **b** Open-circuit voltage of the L-L TENG. **c** The electric output variation of the L-L TENG with a load change. **d** The relationship between the power of the L-L TENG and the load resistance. **e** The charging curves of commercial capacitors of 47 μF, 100 μF, and 330 μF using the L-L TENG. **f** Repetitive cycle test results for the L-L TENG over 9800 repetitions. **g** The relationship between the output current signal of the L-L TENG and the DEX concentration when the PEG concentration was constant. **h** The output performance of the L-L TENG under different environmental humidity values. **i** The output performance of the L-L TENG under different environmental temperatures.

In order to characterize the output performance of the PEG-DEX L-L TENG, a series of electrical signals were tested systematically. As shown in Fig. 4a, b, the open-circuit voltage and closed-circuit current could reach 0.47 V and 720 nA, respectively. The relationship between the output signal and load resistance of the circuit was systematically studied. As shown in Fig. 4c, as the load resistance increased, the voltage showed an increasing trend, while the current showed the opposite relationship.

We used the formula P = UI to obtain the instantaneous power curve and DC equivalent power curve, where the instantaneous power and DC equivalent power at maximum load reached 100 nW and 26.4 nW, respectively, as shown in Fig. 4d. At the maximum instantaneous power density, the relative load voltage could reach 0.19 V, and the relative load current could reach 540 nA. Figure 4 shows the power density diagram, in which the instantaneous power density and DC equivalent power density at the maximum load reached 0.06 W/m^2^ and 0.02 W/m^2^, respectively. The lower voltage in our analysis may have been due to the fact that the L-L TENG was composed of two highly conductive liquid materials. Therefore, the resistance of the L-L TENG for one contact-separation cycle was tested, as shown in Supplementary Fig. 6. It can be seen that the resistance during liquid–liquid contact was ~15 MΩ. When the contact area reached the maximum, the resistance decreased and reaches the minimum value, while the resistance increased slowly when the upper droplet was raised. When the critical point was exceeded, the two droplets separated, and the resistance became infinite. Compared with the other L-L TENGs in Supplementary Table 2, the contact-separation mode showed a superior output performance and stability. When the external load was the same as the inherent impedance of the TENG^57,58^, the maximum power transfer occurred, which provided a certain theoretical basis for practical applications. In addition, the pulse energy of the TENG could be stored in a capacitor. Figure 4e shows that commercial capacitors of 47 μF, 100 μF, and 330 μF could be charged to 0.34 V, 0.25 V, and 0.16 V, respectively, at an ambient humidity of ~45% and room temperature.

In practical applications, the long-term stability/reliability of a nanogenerator is a very important issue. Under the same operating conditions, the output performance of the L-L TENG was tested for more than 9000 repetitions. As shown in Fig. 4f, the closed-circuit current did not decrease, which showed the good stability and durability of the L-L TENG. In order to evaluate the environmental adaptability of the L-L TENG, its outputs with different PEG-DEX concentrations (8–10%, 10%, 12%) and under different ambient humidity and temperature values were tested. As shown in Fig. 4g, as the DEX concentration decreased, the closed-circuit current of the L-L TENG gradually decreased. This may have been related to the increases in the DEX surface tension and viscosity. For a traditional nanogenerator, the environmental humidity can weaken the triboelectric effect and shorten the storage time of the triboelectric charge on the surface. However, according to Fig. 4h, the closed-circuit current of the L-L TENG did not change significantly under different environmental humidity conditions. This was because of the incompatibility between DEX and water. The attachment of micro-droplets to DEX droplets did not change the DEX surface concentration. On the contrary, these could infiltrate the surface of the droplets to prevent the water from becoming volatile and solidifying it, which helped to prolong the life of the L-L TENG. This made the L-L TENG work better in a humid environment than in traditional nanogenerators. Figure 4i shows that the closed-circuit current of the L-L TENG decreased in proportion to the increase in temperature. This was because as the temperature increased, the viscosity of the droplet decreased, and the integrity of the interface decreased, resulting in a decrease in the output performance. In order to exclude the possible contact electrification of the bottom aluminum foil electrode with the bottom droplet, the closed-circuit currents of two different cases were recorded during contact electrification, one with a change in the contact area between the bottom droplet and bottom electrode, and the other without, as shown in Supplementary Fig. 5. It can be clearly seen that the closed-circuit currents of the two were not significantly different, which indicated that the bottom electrode had little effect on the electrical output during the operation of the L-L TENG.

**Fig. 5:**
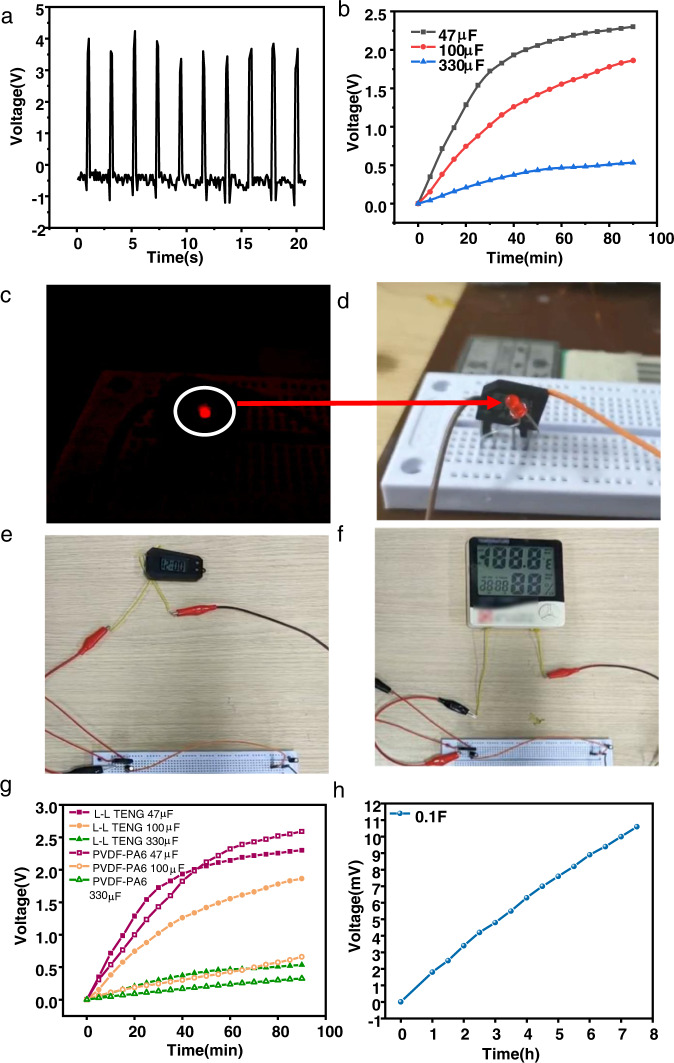
Applications of the L-L TENG. **a** Open-circuit voltage generated by 10 L-L TENGs connected in series. **b** Charging curves for commercial capacitors of 47 μF, 100 μF, and 330 μF. **c**, **d** The series of L-L TENGs could directly light an LED. The (**e**) electronic meter and (**f**) hygrometer could be powered by the series of L-L TENGs. **g** Charging curves for commercial capacitors of 47 μF, 100 μF, and 330 μF using L-L TENG and S-S TENG and (**h**) charging curve for supercapacitor of 0.1 F using L-L TENG.

In order to further verify the ability of the proposed L-L TENG as an energy-generating device to supply power to small portable smart electronic devices, ten groups of liquid droplets were connected in series. As shown in Fig. 5a, the open-circuit voltage could reach 3.92 V after the series connection. Similarly, commercial capacitors of 47 μF, 100 μF, and 330 μF could be charged to 2.3 V, 1.9 V, and 0.5 V, respectively, as shown in Fig. 5b. The L-L TENG was superior to traditional solid TENGs in relation to both a shorter charging period and ability to charge larger capacitors, as shown in Fig. 5g. As shown in Fig. 5c, d, the L-L TENGs connected in series could directly light up a single LED under dark conditions (Supplementary Movie 3). At the same time, using capacitors and rectifiers, the L-L TENG could also power the electronic meter (Supplementary Movie 4), as shown in Fig. 5e, f. This showed the potential for the L-L TENG to be used in the field of portable self-powered equipment. Meanwhile, the large charge density of the L-L TENG could be utilized to charge commonly used power sources such as supercapacitors. Here, a 0.1 F supercapacitor was charged for 7.5 h, and the charging curve was recorded by testing the voltage. As shown in Fig. 5h, the voltage of the supercapacitor reached 10.6 mV, and it could be further charged with a longer charging time or more ATPS droplets.

**Fig. 6:**
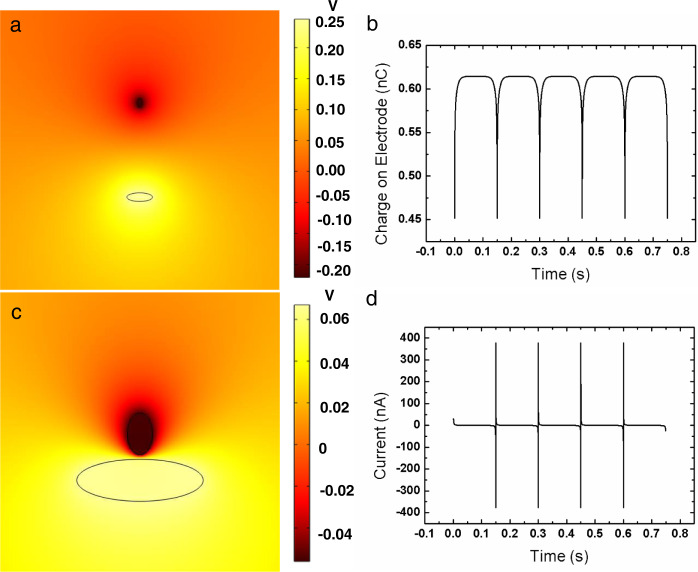
Finite element analysis of the L-L TENG. Electric field distribution simulated by finite element analysis, from far enough (**a**) to close enough (**c**). **b** The induced electric current on the electrode over time. **d** Current waveform obtained by charge waveform differential.

To understand the details of the L-L TENG, a COMSOL finite element analysis was performed. According to the basic principle of a TENG, the Maxwell–Poisson equation is used to describe the relationship between the displacement field (**D**) and charge density (*ρ*):

1 $${{{{{\boldsymbol{\nabla }}}}}}\bullet {{{{{\bf{D}}}}}}=\rho,$$

where *ρ* represents the charge density, and the space charge density, *ρ*, is zero here. The relationship between the displacement field (**D**) and electric field (**E**) in an isotropic medium are described by the following equation:

2 $${{{{{\bf{D}}}}}}={\varepsilon }_{r}{\varepsilon }_{0}{{{{{\bf{E}}}}}},$$

where ${\varepsilon }_{r}$ and ${\varepsilon }_{0}$ represent the dielectric constants of the dielectric material and vacuum, respectively. In the absence of a magnetic field, the electric field (E) can be derived as follows:

3 $${{{{{\bf{E}}}}}}=-{{{{{\boldsymbol{\nabla }}}}}}\varphi,$$

where *φ* represents the electrical potential, and the relationship between the electrical potential and charge density can be deduced as follows:

4 $$-{\varepsilon }_{r}{\varepsilon }_{0}{{{{{{\boldsymbol{\nabla }}}}}}}^{2}\varphi=\rho,$$

where space charge density *ρ* is also zero here. The above formulas could be used to calculate the stationary space potential, as shown in Fig. 6a, c, and it could clearly be seen that during the contact-separation process, the electrical potential decayed with distance, and the voltage difference between the electrodes could be enhanced by increasing the gap distance between the two droplets. Meanwhile, the charge on the back electrode could also be simulated accordingly, showing an exponential approach to a constant, as shown in Fig. 6b. This is easily understood. When the triboelectric charge separated far enough, the two droplet–electrode pairs could be regarded as individuals, forming two capacitors, where each capacitor had two electrodes with equivalent charge and different signs. The current waveform could then be derived from the charge waveform differential, and the result is shown in Fig. 6d. It is worth noting that the simulation was still limited as a result of a lack of accurate duration times for the contact and separation, which were difficult to obtain because of the shape change of the TENG materials. Future directions for improving the simulation model may include considering the contributions of micro-surface contact details to the induced displacement current and equivalent capacitance.

### Biocompatibility analysis

**Fig. 7:**
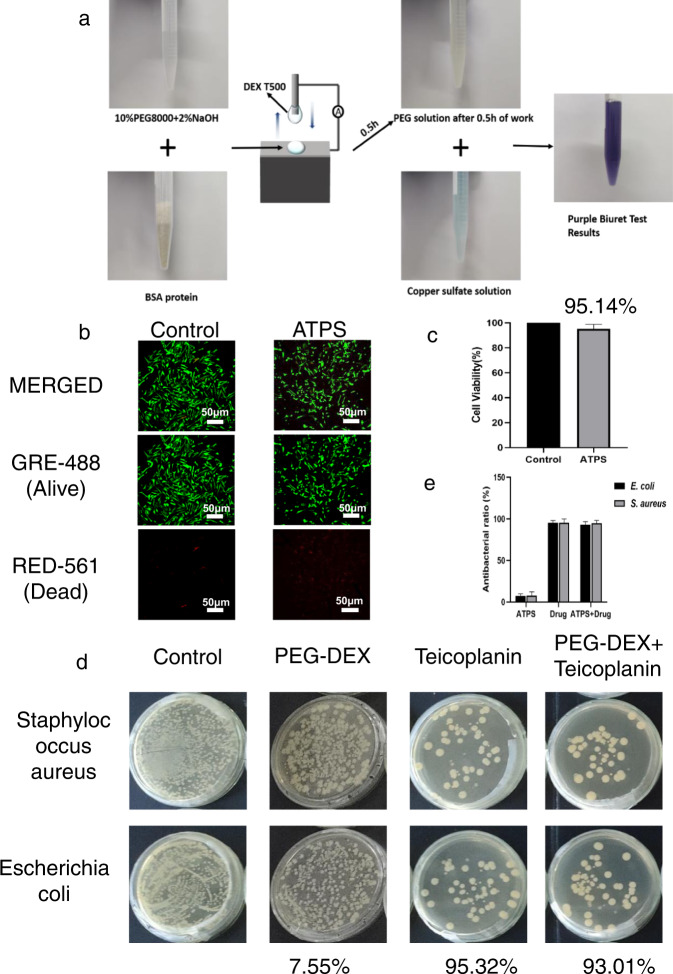
Superior biocompatibility of the L-L TENG as the microreactor and drug carrier. **a** PEG droplet with BSA protein added to the 1% CuSO_4_·5H_2_O solution before and after the L-L TENG operated for 0.5 h. **b** Live/dead cell fluorescence images. **c** The proportions of living cells in (**b**). **d** Antibacterial experiment with PEG-DEX system loaded with antibacterial drugs. **e** Histogram of the antibacterial rate of antibacterial experiment in (**d**).

An ATPS is widely used for the separation, purification, and enrichment of biomolecules^44,46,59,60^. Making full use of the morphology control ability of this system can lead to the development of liquid electronic devices. Thus, a biocompatibility analysis was valuable for its future use in implantable devices. In order to verify the biocompatibility of the proposed L-L TENG, a biuret assay and cytotoxicity experiments were conducted. A small amount of BSA protein solution was added to the PEG droplet of the L-L TENG. After the L-L TENG operated for 0.5 h, the protein-mixed PEG droplet was dropped into a 1% CuSO_4_·5H_2_O solution (Supplementary Movie 5). The original light blue CuSO_4_ solution appeared obviously purple after a period, as shown in Fig. 7a. This indicated that the BSA protein was not damaged either in the PEG droplet or during the contact-separation process, demonstrating the good biocompatibility of the L-L TENG for biomolecules. Moreover, the cytotoxicity of a material itself is another important indicator to evaluate its biocompatibility. The cytocompatibility of the PEG-DEX was investigated using a living/dead assay. As illustrated by the fluorescence images in Fig. 7b, the number of living cells (green) cultured with the PEG-DEX system was not significantly reduced, and the cell morphology was stable compared to the blank control group. Moreover, there were almost invisible red signals in the microscopic field of view, indicating a particularly low number of dead cells during the culture process. In addition, the cytotoxicity of the PEG-DEX system was evaluated qualitatively by conducting an MTT assay. According to the cell viability, cytotoxicity results can be divided into three levels (<50%, toxic; 51–70%, low cytotoxicity; >70%, non-toxic). As shown by the plots in Fig. 7c, the cell viability of the PEG-DEX system was ~95.14%, indicating that the PEG-DEX system was obviously non-cytotoxic. In summary, the PEG-DEX system had superior cytocompatibility and biological safety, and could be used as medical materials.

An ATPS can gain versatility by loading functional materials. Thus, the drug-loading performance was investigated to show the development potential of the L-L TENG. *Staphylococcus aureus* (*S. aureus*) and *Escherichia coli* (*E. coli*) were selected as Gram-positive bacteria and Gram-negative bacteria for the bacteriostatic test, respectively. As shown by the optical images of the bacterial colonies and plots in Fig. 7d, e, respectively, the PEG-DEX system showed no obvious antibacterial activity against *S. aureus* and *E. coli.* Teicoplanin is a recognized antibiotic agent, and its antibacterial ratios against *E. coli* and *S. aureus* are 95.32% and 95.19%, respectively. Compared with teicoplanin, the teicoplanin-loaded PEG-DEX system had similar antibacterial properties, demonstrating the great potential of the PEG-DEX system for future antibacterial applications. In summary, the proposed L-L TENG not only had excellent biocompatibility, but could also load antibacterial drugs as a demonstration of its potential in the development of future designs. This will be valuable for systems that are applicable to living organisms.

A vertical contact separation L-L TENG was designed using an immiscible ATPS, and a similar repeatable contact-separation behavior was achieved. The actual output power of this L-L TENG could reach 0.06 W/m^2^ (DC equivalent power of 0.02 W/m^2^). The maximum output charge of a single droplet was determined to be 129 nC (charge density 1.81 × 10^−2^ C/m^2^) and 16 nJ per cycle. The charge density could be attributed to the higher effective contact area of the immiscible aqueous–aqueous interface^22^. The output performance of this nanogenerator did not decrease in a humid environment. It had good biocompatibility, and could be conveniently loaded with drugs, which would allow it to be used for applications such as implantable electronic devices. In the future, a higher output energy density could be obtained by designing a quicker L-L TENG.

## Methods

### Materials

The raw materials included polyethylene glycol (PEG, Mw 8000, Sigma-Aldrich Chemical Co., St. Louis, USA), dextran (DEX Mw 500,000, Shanghai Ryon Biological Technology Co. Ltd, Shanghai, China), copper sulfate pentahydrate (Aladdin Chemistry Co. Ltd, Shanghai, China), bull serum albumin (BSA protein) (Sigma-Aldrich Chemical Co., St. Louis, USA), teicoplanin (Aladdin Chemistry Co. Ltd, Shanghai, China), phosphate buffered saline solution (PBS, Aladdin Chemistry Co. Ltd, Shanghai, China), methyl thiazolyl tetrazolium (MTT, Aladdin Chemistry Co. Ltd, Shanghai, China), rhodamine B (Aladdin Chemistry Co. Ltd, Shanghai, China), and sodium hydroxide (NaOH, Sinopharm Chemical Reagent Co., Ltd. Shanghai, China). These were used without further purification. RPMI-1640 Medium and a Calcein-AM/PI living/dead cell double staining kit were purchased from Beijing Solarbio Science & Technology Co., Ltd. L929 fibroblasts were purchased from Sigma­Aldrich Chemical Co.

### PEG and DEX droplet acquisition method

An appropriate amount of PEG was weighed and dissolved in 50 g of deionized water, and then an appropriate amount of DEX was slowly added in batches. After the DEX was completely dissolved, the entire solution sample was allowed to stand for 12 h, and obvious layering occurred. The upper layer was the PEG-rich solution, and the lower layer was the DEX-rich solution. The two solutions were separately removed for further use.

### Material characterization

The infrared absorption spectra of the PEG and DEX powders, solutions, and dried powders were obtained using Fourier transform infrared spectroscopy (FTIR, Thermo Scientific Nicolet iN10). The viscosity and interfacial tension values of the PEG-rich and DEX-rich solutions were measured using a spinning drop tensiometer (KRUSS GmbH, Germany) and rolling ball viscosimeter (Haake-GmbH, Germany), respectively. The contact and separation states of the PEG-rich and DEX-rich solutions were recorded using a digital microscope.

The output current signal of the L-L TENG was recorded by a picoammeter (Keithley 6517), current amplifier (SR 570), and digital oscilloscope (Gwinstek, GDS-2102). The output voltage signal was recorded by a digital oscilloscope (Gwinstek, GDS-2102). The charging voltage of the capacitor was measured by a digital multimeter (Rigol DM 3058).

### Biocompatibility

#### Cytotoxicity

The cytotoxicity of the PEG-DEX system was evaluated by culturing L929 cells. The L929 cells were seeded into 96-well plates with 1 × 10^4^ cells per well and cultured in 100 μL of RPMI-1640 medium for 24 h. The PEG-DEX system was dissolved in RPMI-1640 medium to prepare the sample medium. The sample medium was then filtered with a membrane (0.22 μm) to ensure sterility. Subsequently, cells were respectively incubated with normal medium as a control group and sample medium as a test group for 48 h in 96-well plates. After washing the plates with PBS, MTT (5 mg/mL) in RPMI-1640 (10%) medium was added to each well. After 4 h of incubation, each well was washed with PBS to remove the old medium, and DMSO was added to dissolve formazan crystals. The absorbance of every well was read at 570 nm with a microplate. Each process was repeated three times. The cell viability was calculated as follows:

5 $${{{{{\rm{Cell}}}}}}\,{{{{{\rm{viability}}}}}}\left(\%\right)=\frac{{{OD}}_{{test}}}{{{OD}}_{{control}}} \times 100\%.$$

### Fluorescence staining of living cells

The Calcein-AM/PI living/dead cell double staining kit was used for a living and dead cell level analysis. In brief, L929 cells were inoculated in a 24-well plate (2 × 10^4^ cells/well). Then, the prepared sample medium and normal medium were injected into the wells containing L929 and incubated for 24 h. After washing with PBS, the L929 was stained in a mixture of Calcein-AM (2 μM) and PI (4.5 μM). Finally, the state of the cells was observed using fluorescence microscopy.

### Antibacterial activity

*Staphylococcus aureus* (*S. aureus*) and *Escherichia coli* (*E. coli*) were selected as different Gram-positive and Gram-negative model microorganisms to carry out antimicrobial tests, respectively. First, a bacterial suspension of 1 × 10^4^ CFU/mL was prepared with sterile PBS. Then, 5 ml of the bacterial suspension was co-cultured with the PEG-DEX system and teicoplanin at 37 °C for 4 h. A bacterial suspension without the PEG-DEX system was used as the control group. Next, 100 μL of the bacterial suspension was added to the surface of solid agarose medium and spread evenly. After incubation at 37 °C for 24 h, the colony units on the solid agarose were observed and recorded. The antibacterial ratio was calculated using the following formula:

6 $${{{\rm{Antibacterial}}}} \, {{{\rm{Ratio}}}} (\% )=\frac{{N}_{b}-{N}_{s}}{{N}_{b}} \times 100\%,$$

where *N*_*b*_ is the number of bacterial colonies in the control group, and *N*_*s*_ is the number of bacterial colonies in the samples.

## Supplementary information

Supplementary Information

Description of Additional Supplementary Files

Supplementary Movie 1

Supplementary Movie 2

Supplementary Movie 3

Supplementary Movie 4

Supplementary Movie 5

## Data Availability

The data that support the findings of this study are available within the text, including the Methods and Supplemental information. Raw datasets related to the current work are available from the corresponding author on reasonable request.
