# Supplementary material for: Liquid-liquid triboelectric nanogenerator based on the immiscible interface of an aqueous two-phase system
Source: Nat Commun. 2022 Sep 9;13:5316. doi: 10.1038/s41467-022-33086-2 (PMC9463141; doi:10.1038/s41467-022-33086-2)
Supplement: Supplementary file 1 — Supplementary Information [file 41467_2022_33086_MOESM1_ESM.pdf]

Supplementary Informations for

Liquid-liquid triboelectric nanogenerator based on the immiscible interface of an aqueous two-phase system

Lu Ye[1,2,4,5,6†], Jiang Longlong[1,2,4,5,6†], Yu Yang[1], Wang Dehua[1], Sun Wentao[3], Liu Yang[7,8], Yu Jing[9], Zhang Jun[1], Wang Kai[10], Hu Han[11], Wang Xiao[12], Ma Qingming[2*], Wang Xiaoxiong[1,4,5,6*]

[1.]College of Physics, Qingdao University, Qingdao 266071, China

[2.]School of Pharmacy, Qingdao University, Qingdao 266071, China

[3.]School of Health and Life Sciences, University of Health and Rehabilitation Sciences, Qingdao 266113, China

[4.]University-Industry Joint Center for Ocean Observation and Broadband Communication, College of Physics, Qingdao University, Qingdao 266071, China

[5.]Weihai Innovation Research Institute of Qingdao University, Weihai 264200, PR China

[6.]Collaborative Innovation Center for Eco-Textiles of Shandong Province, and State Key Laboratory of Bio-Fibers and Eco-Textiles, Qingdao University, Qingdao 266071, China

[7.]Microsystem and Terahertz Research Center, China Academy of Engineering Physics, Chengdu 610200, China

[8.]Institute of Electronic Engineering, China Academy of Engineering Physics, Mianyang 621999, China

[9.]School of Physics and Electronics, Shandong Normal University, Jinan 250014, China

[10.]School of Electrical Engineering, Qingdao University, Qingdao 266000, China

[11.]State Key Laboratory of Heavy Oil Processing, College of Chemical Engineering, China University of Petroleum (East China), Qingdao 266580, China

[12.]School of Mathematics and Statistics, Qingdao University, Qingdao 266071, China

[†]These authors contributed equally to this work.

*Corresponding authors: wangxiaoxiong69@163.com and maqm2012@hotmail.com

Table of contents

Supplementary Note 1: The description of the originality of our developed L-L TENG.

Supplementary Note 2: The description of Finite Element Simulation.

Supplementary Figure 1 to Supplementary Figure 9.

Supplementary Table 1 to Supplementary Table 2.

Supplementary References

Supplementary Note 1

Description of the originality of our developed L-L TENG

The originality of our work is based on the recoverable immiscible aqueous-aqueous interface (IAAI) composed by PEG-DEX ATPS and lies in three coordinated parts:

I. the first realization of reversible liquid-liquid contact electrification that can truly realize energy output in conventional energy harvesting systems, demonstrating the possibility of developing new type of L-L TENGs that can perform as reversible power sources in common scenarios.

II. the first successful interdisciplinary intersection between ATPS and nanogenerators, inspiring new ways to develop novel types of electronic devices, enriching the research connotation of liquid-liquid phase separation (LLPS), and opening up new research areas of great significance in multiple basic and applied disciplines.

III. the first demonstration of addressing the long-standing problem of over-approximation of simulations in the field of nanogenerators and providing important reference for developing more accurate simulation of nanogenerators in the future.

Detailed descriptions are shown below:

(Part I) We have done a thoroughly literature review about the current existing L-L TENGs reports and only four published works can be found:

① "Power generation from the interaction of a liquid droplet and a liquid membrane (Nature Communications, Vol. 10, 2019)"

② "Studying of contact electrification and electron transfer at liquid-liquid interface (Nano Energy, Vol. 87, 2021)"

③ "Quantifying Contact-Electrification Induced Charge Transfer on a Liquid Droplet after Contacting with a Liquid or Solid (Advanced Materials Vol. 33 2021)"

④ "Non-contact and liquid–liquid interfacing triboelectric nanogenerator for self-powered water/liquid level sensing (Nano Energy, Vol. 72, 2020)"

The output efficiencies of all the published L-L TENGs are relatively low, and not applicable to be used as reversible power source in common scenarios, which can be achieved in our work, performing as a truly renewable energy; and the working principles of these L-L TENGs are indeed different from that of our work. Moreover, net electric output of L-L TENG in contact-separation mode has never been reported, making our L-L TENG the first one in this area.

There are usually five modes of TENGs, including contact-separation mode, lateral sliding mode, single-electrode mode, freestanding mode and electrostatic induction mode "Triboelectric Nanogenerator: A Foundation of the Energy for the New Era (Advanced Energy Materials Vol. 9 2019)". We have done a thoroughly literature review about the contact-separation mode TENGs between liquid-liquid contact materials. Only one published work based on contact separation electrification (work ③) can be found, without reporting net energy output, showing that we are the original report of contact-separation mode L-L TENG. The reason why no net energy output was reported in their published work can be attributed to the additional energy requirement to drive the ultrasonic suspension system at magnitude of W, and the resultant L-L electrification case cannot be applied as power source in practical applications.

(Part II) The immiscibility between oil and water is long-known. Without enough material choice, the development of L-L TENG is limited. Based on our work, more immiscible interfaces can be

developed far beyond the limited system, triggering fast developing of L-L TENGs. ATPS is an emerging technology which has attracted widespread scientific interests due to its great potentials in multi-disciplinary fundamental researches and biomedical applications. However, it has not yet been used in the field of L-L TENG and leaves large gaps for realizing its potentials in developing electronic devices. Particularly, ATPS-based electronics can offer unique all-aqueous interface that is highly relevant to the electrical sensing and signal transmission of living organisms, therefore can establish the foundation of aqueous electronic devices for investigating the fundamentals of various electrical phenomena in life, which cannot be achieved by any existing L-L TENG. Thus, our work of ATPS-based L-L TENG would offer great potentials in future development of aqueous electronic devices. In addition, the efficient reversible contact-separable PEG-DEX ATPS for triboelectric nanogenerators in our work is also a material science discovery which will offer reference for researchers on electro-chemistry, electro-biology and electronics. Further, as one representative liquid-liquid phase separation (LLPS), PEG-DEX ATPS has recently attracted significant scientific interests in multi-disciplines, ranging from the revelation of the origin of life to the analysis of fundamentals for various pathological disordered diseases. Therefore, our successful interdisciplinary intersection between ATPS and nanogenerators will enrich the research connotation of LLPS and inspire research areas of great significance in multiple basic and applied disciplines.

(Part III) There is a long-standing problem of over-approximation of simulations in the field of nanogenerators, which typically leads to the discrepancies between numerical model and experimental results. Here in our work, we have systematically investigated the reasons of the discrepancies and offered an alternative. Discrepancies between numerical model and experiments were commonly due to the inconsistency between the stationary simulation and the dynamic test results. In the actual test, the working principle of the voltmeter is to amplify and measure the current flowing through the known resistance, and obtain the voltage value by multiplying the small current by the resistance. The test results are similar to closed-circuit states, and therefore require dynamic calculations based on charge transfer rather than static potential decay. In fact, static potential decay is the mainstream simulation idea in the field of nanogenerators. Here dynamic simulation was used, and the obtained voltage and current signals that are highly consistent with the experimental results, solving the long-standing problem of over-approximation in simulations in the field of nanogenerators. This provides an important reference for the simulation of nanogenerators in the future. Additionally, this update also increases the originality of this work, providing important reference for developing more accurate simulation of nanogenerators in the future.

Supplementary Note 2
Finite Element Simulation

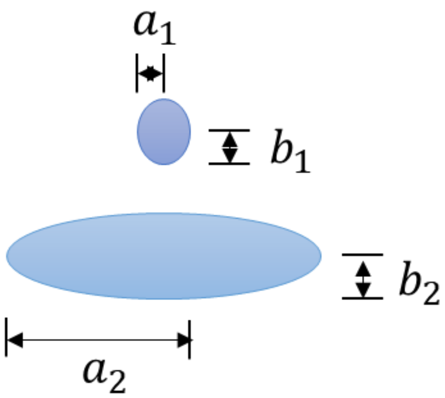

Supplementary Figure 1. Geometry settings of the simulation.

COMSOL has been widely used in related analysis such as potential simulation of nanogenerators. In this paper, the typical sizes of the upper droplets we set are: a_1=0.3 mm, b_1=0.5 mm, and the typical sizes of the lower droplets are: a_2=1.5 mm, b_2=0.5 mm. The surface charge density of the droplet is set 1.8×10^{-2} C/m^2 as a boundary condition, according to the experimental results. After assigning the initial charge amount after the contact electrification, changes in the potential and charge accumulation on the electrodes are separately explored to simulate the case of open-circuit and closed-circuit.

To simulate the actual situation, the current waveform should be simulated first, because the voltage data can be obtained by the current data multiplied by the corresponding resistance, considering that the mainstream voltage meter works on this mechanism. Current calculation requires differentiation of the electrode charge. The initial electrode charge is shown in **Supplementary Fig. 2**(a), which can be easily interpolated by fitting this data. The periodic change diagram of electrode charge is shown in **Supplementary Fig. 2**(c), which can be obtained by calibrating the droplet motion state expressed by function as shown in **Supplementary Fig. 2**(b) as a variation of time to the parametric scanning result of the charge on electrode by interpolating the time dependent points with the fitting formula. At this time, the actual current data can be obtained by differentiating the data diagram, as shown in **Supplementary Fig. 2**(d). The actual voltage data is the current data multiplied by the system resistance, and the results are shown in **Supplementary Fig. 2**(e). In most test cases, the waveform will be asymmetric, and here the separation duration is elongated and asymmetric current waveform will be obtained, as shown in **Supplementary Fig. 2**(f).

Supplementary Figure 2. Interpolation process of finite element analysis results.

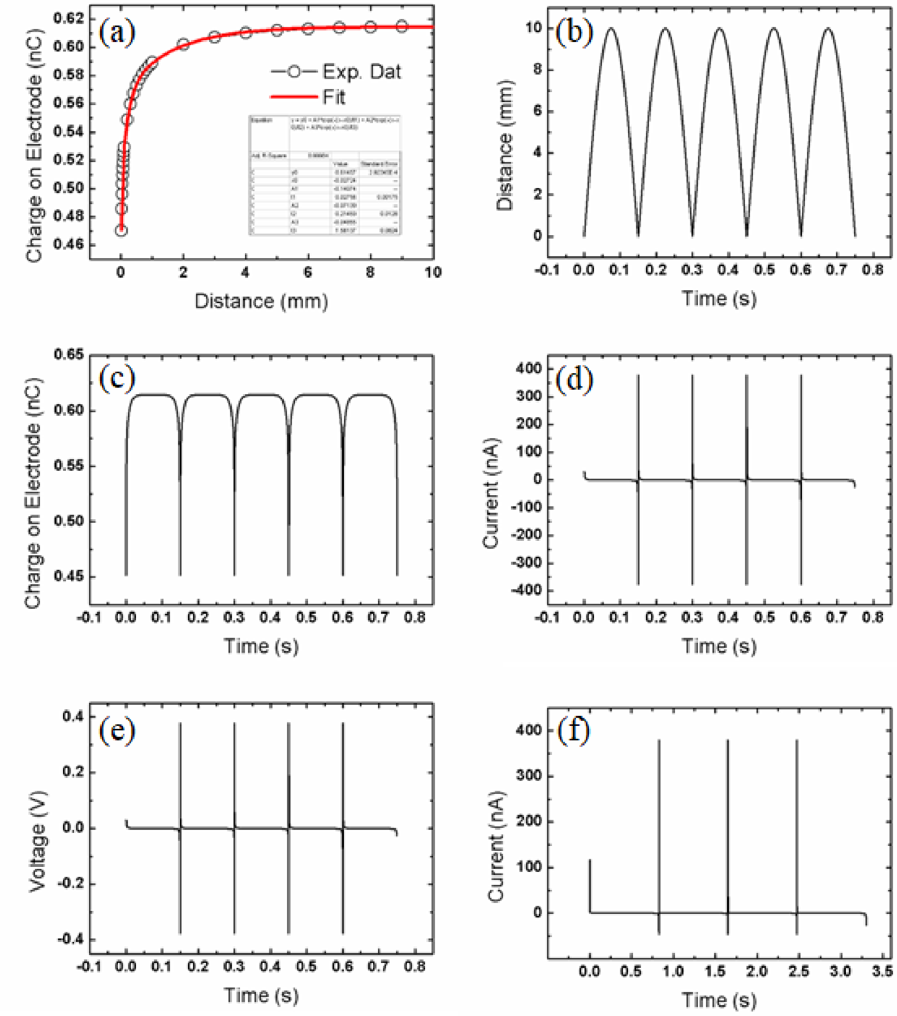

Supplementary Figure 2: (a) Electrode charge variation with distance. (b) Motion waveform of the moving platform. (c) Cycled electrode charge waveform. (d) Differential current obtained from the charge waveform and (e) voltage waveform obtained from current waveform. (f) Asymmetric waveform obtained by elongating the separation duration.

Supplementary Figure 3. Infrared spectrum.

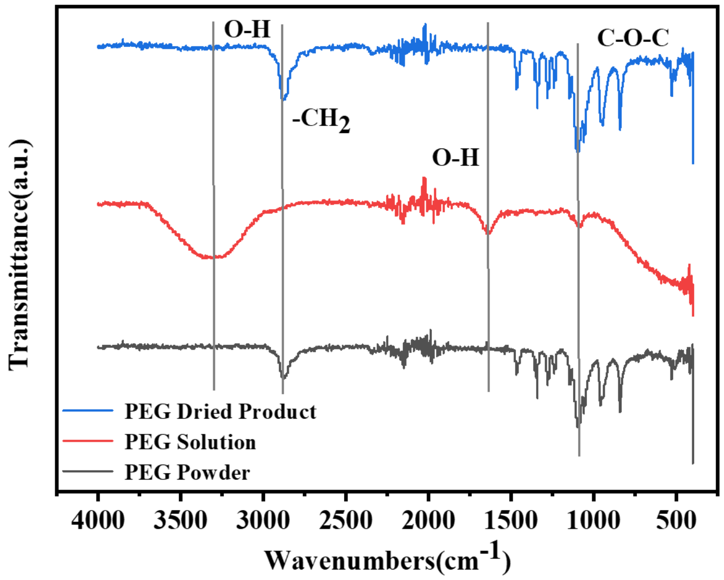

Supplementary Figure 3a: The infrared spectrum of commercial PEG, the PEG aqueous solution in the PEG-DEX system and the PEG after the PEG aqueous solution in the PEG-DEX system is dried to remove water.

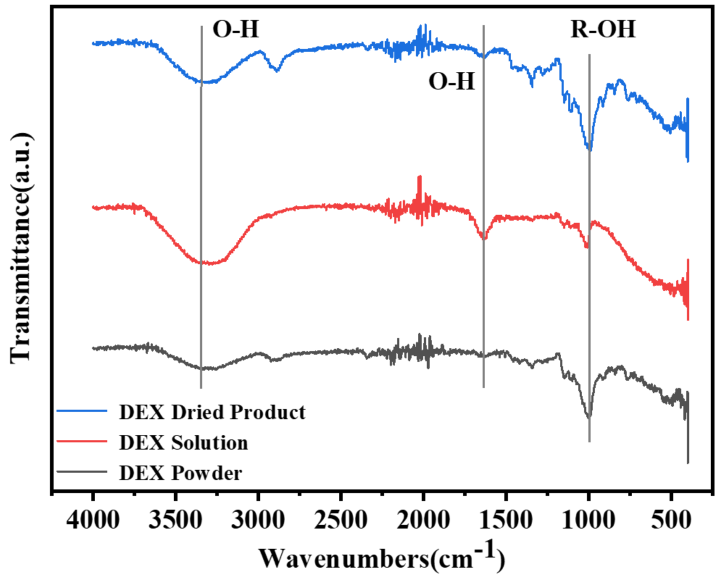

Supplementary Figure 3b: The infrared spectrum of commercial DEX, the DEX aqueous solution in the PEG-DEX system and the DEX after the DEX aqueous solution in the PEG-DEX system is dried to remove water.

Supplementary Figure 4. Closed-circuit current waveforms comparison between various situations.

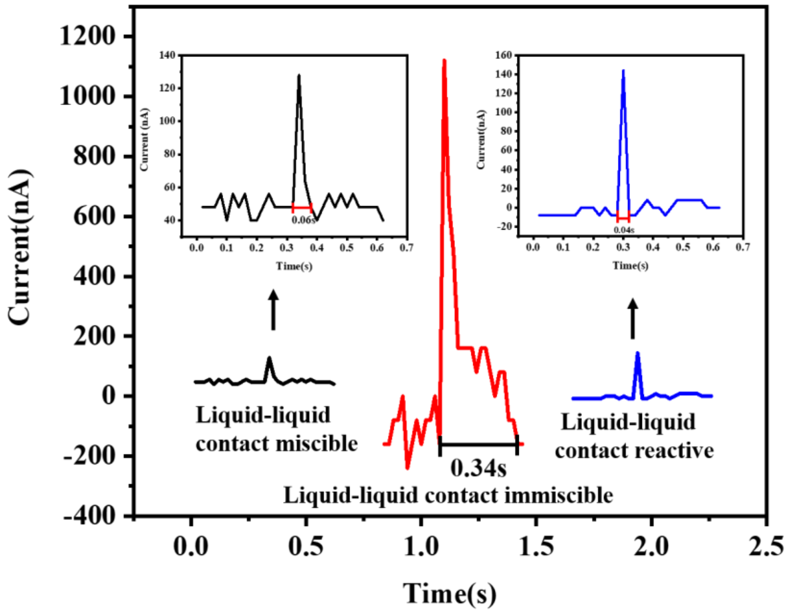

Supplementary Figure 4: The closed-circuit current waveforms of liquid-liquid contact immiscible(PEG-DEX), liquid-liquid contact miscible (Sodium citrate-Na_2CO_3) and liquid-liquid contact reactive ($CaCl_2$-Na_2CO_3).

Supplementary Figure 5. Exclusion of the affection from electrode.

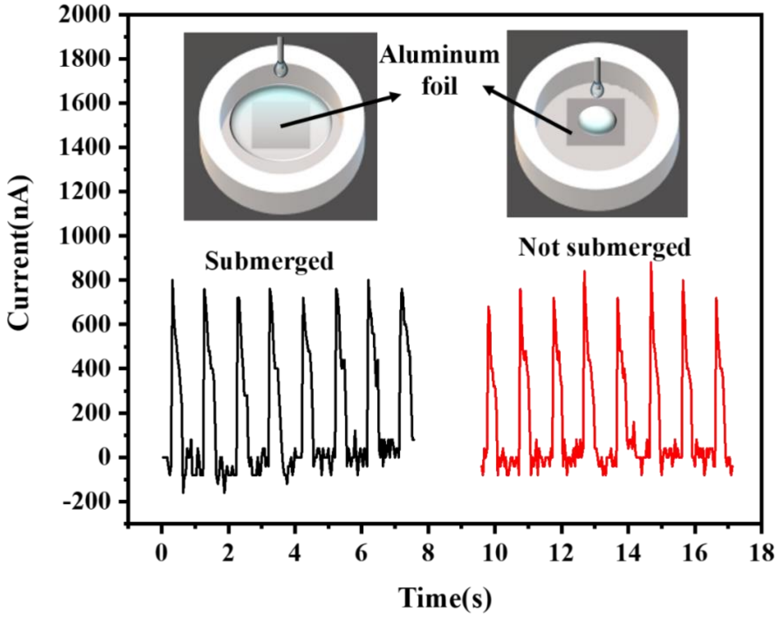

Supplementary Figure 5：Closed-circuit current comparison of the PEG solution with submerged bottom electrode and droplet on bottom electrode.

Supplementary Figure 6. Variation of resistance during contact separation.

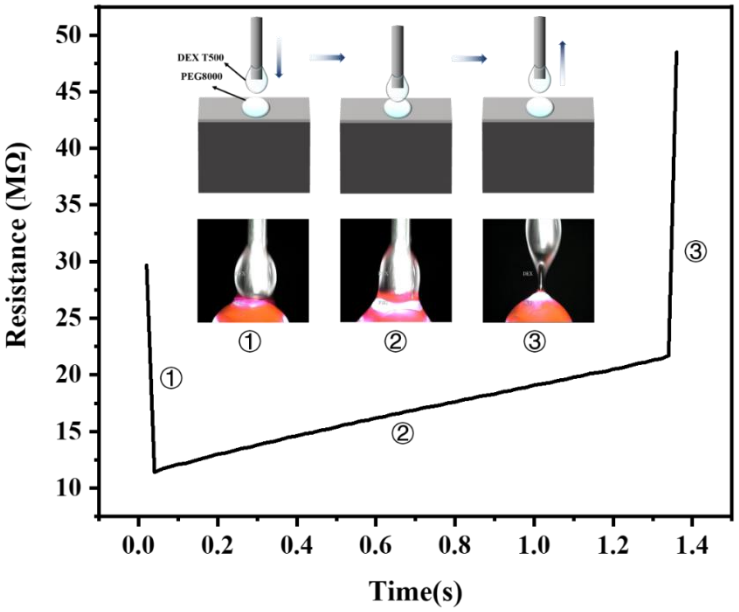

Supplementary Figure 6: Variation of resistance during contact separation.

Supplementary Figure 7. The relationship between the power density of the L-L TENG and the load resistance.

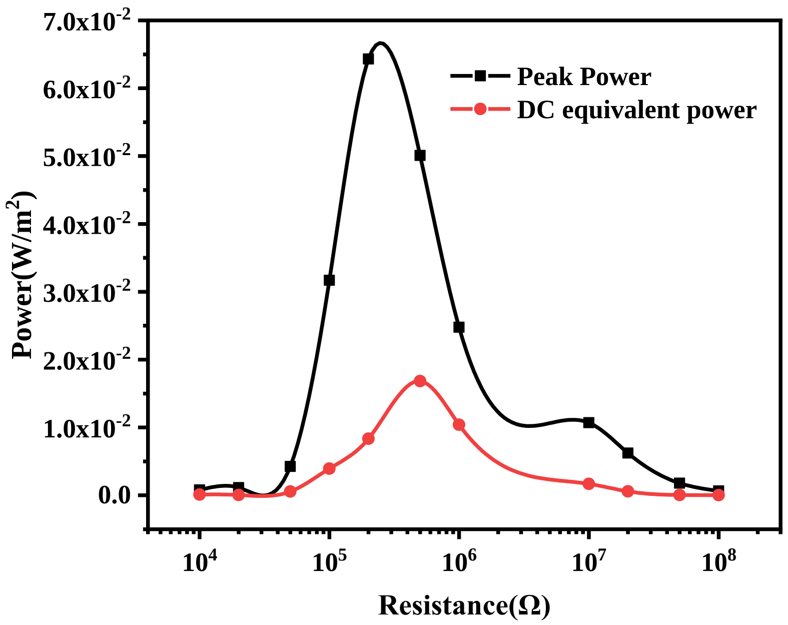

Supplementary Figure 7: The relationship between the power density of the L-L TENG and the load resistance.

Supplementary Figure 8. Charge analysis of the L-L TENG.

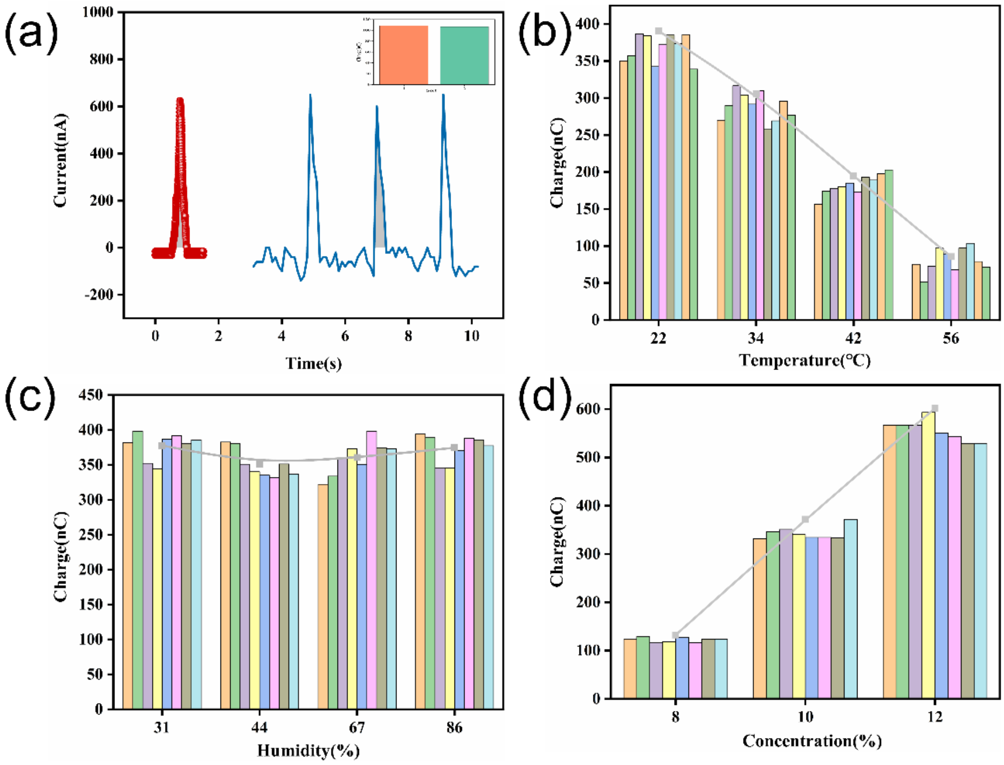

Supplementary Figure 8: (a) Individual pulse with different sampling frequency and insert showed the comparison between the integration of the two forms of peaks. (b) The amount of charge transferred by a single pulse at different temperatures. (c) The amount of charge transferred by a single pulse at different humidity. (d) The amount of charge transferred by a single pulse at different concentrations.

Supplementary Figure 9. Energy analysis of the L-L TENG from voltage waveform.

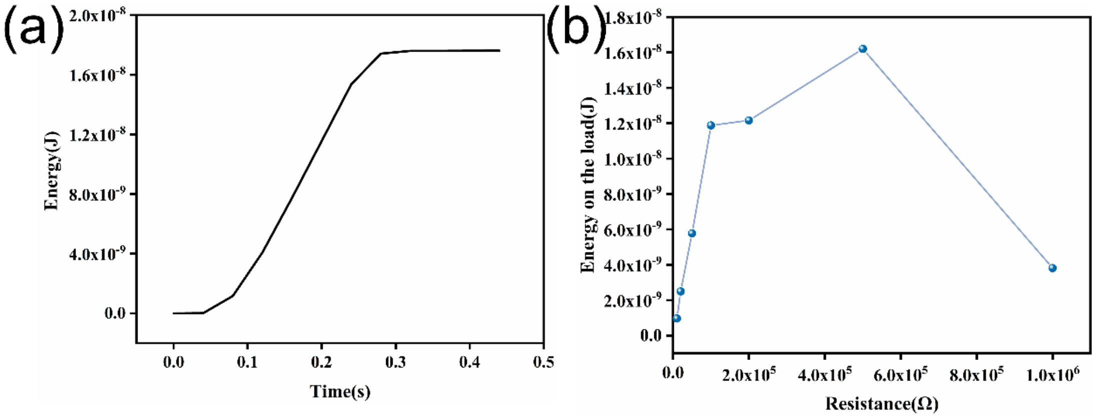

Supplementary Figure 9: (a) Amount of electrical energy generated from a single droplet in a single cycle of contact-separation and (b) Energy generated on different loads.

Supplementary Tables 1: Contact output between different aqueous droplets.
“-” means the output current is too small to be recorded.

Friction material	Liquid metal	Paraffin	Deionized water	N-decane	Tetradecane	N-hexadecane	K_3PO_4
Liquid metal	—	—	2 nA	—	—	—	2 nA
Paraffin	—	—	0.2 nA	—	—	—	—
Deionized water	2 nA	0.2 nA	—	0.28 nA	0.28 nA	0.16 nA	Fusion
N-decane	—	—	0.28 nA	—	—	—	—
Tetradecane	—	—	0.28 nA	—	—	0.18 nA	—
N-hexadecane	—	—	0.16 nA	—	0.18 nA	0.18 nA	10 nA
K_3PO_4	2 nA	—	Fusion	—	—	10 nA	—
Glucan	0.5 nA	—	Fusion	—	0.05 nA	—	—
Sodium citrate	—	—	Fusion	—	—	—	—
PEG	0.05 nA	—	Fusion	—	0.05 nA	—	12 nA
$CaCl_2$	2.4 nA	—	Fusion	—	—	—	Produce milky white precipitate
NaA	1.5 nA	—	0.1 nA	—	—	—	Produce milky white precipitate
$NaCO_3$	3.6 nA	—	Fusion	—	—	—	12 nA
Silicone oil	—	—	0.1 nA	—	—	—	—
Friction material	Glucan	Sodium citrate	PEG	$CaCl_2$	NaA	$NaCO_3$	Silicone oil
Liquid metal	0.5 nA	—	0.05 nA	2.4 nA	1.5 nA	3.6 nA	—
Paraffin	—	—	—	—	—	—	—
Deionized water	Fusion	Fusion	Fusion	Fusion	0.1 nA	Fusion	0.1 nA
N-decane	—	—	—	—	—	—	—
Tetradecane	0.05 nA	—	0.05 nA	—	—	—	—
N-hexadecane	—	—	—	—	—	—	—
K_3PO_4	—		12 nA	Produce milky white precipitate	Produce milky white precipitate	12 nA	—
Glucan	—	9 nA	8 nA	11 nA	8 nA	15 nA	—
Sodium citrate	9 nA	—	8 nA	10 nA	8 nA	15 nA	—

PEG	8 nA	8 nA	—	11 nA	9 nA	15 nA	—
$CaCl_2$	11 nA	10 nA	11 nA	—	9 nA	15 nA	—
NaA	8 nA	8 nA	9 nA	8 nA	8 nA	Produce milky white precipitate	—
$NaCO_3$	15 nA	15 nA	15 nA	Produce milky white precipitate	15 nA	15 nA	—
Silicone oil	—	—	—	—	—	—	—

Supplementary Tables 2: Output performance of multiple types of triboelectric nanogenerators.

Friction material	Contact interface	Voltage	Current	Charge	Charge density	Power	Power density
PVDF-PA6[5]	Solid-Solid	164 V	392 nA	—	—	—	120 m W/m^2
Al foil-Kapton[6]	Solid-Solid	12 V	0.25 μA	—	—	—	8.44 m W/m^2
γ-Fe^2 O^3/ PVDF[7]	Solid-Solid	250 V	5 μA	—	—	0.17 mW	0.117 W/m^2
PVDF-HFP -PA6[8]	Solid-Solid	80 V	1.67 μA	32nC	2× $10^{-6}C/m^2$*	0.45 mW	500 m W/m^2*
PTFE- Deionized water[9]	Solid-Liquid	300 V	13.5 μA	—	—	1.03 mW	147 m W/m^2*
FEP- Deionized water[10]	Solid-Liquid	77.0 V	52.0 nA	30.7 nC	2.44× $10^{-3}C/m^2$*	23.3 μW	1.855 W/m^2*
PTFE- Deionized water[1]	Solid-Liquid	143.5 V	270.0 μA	49.8 nC	3.96× $10^{-3}C/m^2$*	629.256uW*	50.1 W/m^2
Kapton- liquid metal[11]	Solid-Liquid	679 V	9 μA	0.645 μC	5.13× $10^{-5}C/m^2$*	84.152uW*	6.7 W/m^2
Liquid droplet - Liquid membrane[2]	Liquid-Liquid	4 V	60 nA	1 nC	7.96× $10^{-5}C/m^2$*	137.4 nW	0.0109 W/m^2*
Ferrofluid - Lubricant oil layer[3]	Liquid-Liquid	1.3 V	1 nA	—	—	—	—
Transformer oil - Falling droplets[4]	Liquid-Liquid	—	40 pA	-5.3 pC	4.21× $10^{-7}C/m^2$*	—	—
This paper works	Liquid-Liquid	0.4 V	700 nA	129 nC	1.81× $10^{-2}C/m^2$	100 nW	0.06 W/m^2

* Calculated from the original data.

Supplementary References

1. Xu W, *et al*. A droplet-based electricity generator with high instantaneous power density. *Nature* **578**, 392-396 (2020).
2. Nie J, Wang Z, Ren Z, Li S, Chen X, Lin Wang Z. Power generation from the interaction of a liquid droplet and a liquid membrane. *Nature Communications* **10**, 2264 (2019).
3. Wang P, Zhang S, Zhang L, Wang L, Xue H, Wang ZL. Non-contact and liquid–liquid interfacing triboelectric nanogenerator for self-powered water/liquid level sensing. *Nano Energy* **72**, 104703 (2020).
4. Zhao X, *et al*. Studying of contact electrification and electron transfer at liquid-liquid interface. *Nano Energy* **87**, 106191 (2021).
5. Wang N, *et al*. Anisotropic Triboelectric Nanogenerator Based on Ordered Electrospinning. *ACS Applied Materials & Interfaces* **12**, 46205-46211 (2020).
6. Zheng Q, *et al*. In Vivo Powering of Pacemaker by Breathing-Driven Implanted Triboelectric Nanogenerator. *Advanced Materials* **26**, 5851-5856 (2014).
7. Fatma B, Bhunia R, Gupta S, Verma A, Verma V, Garg A. Maghemite/Polyvinylidene Fluoride Nanocomposite for Transparent, Flexible Triboelectric Nanogenerator and Noncontact Magneto-Triboelectric Nanogenerator. *ACS Sustainable Chemistry & Engineering* **7**, 14856-14866 (2019).
8. Qin Z, Yin Y, Zhang W, Li C, Pan K. Wearable and Stretchable Triboelectric Nanogenerator Based on Crumpled Nanofibrous Membranes. *ACS Applied Materials & Interfaces* **11**, 12452-12459 (2019).
9. Zhao XJ, Kuang SY, Wang ZL, Zhu G. Highly Adaptive Solid–Liquid Interfacing Triboelectric Nanogenerator for Harvesting Diverse Water Wave Energy. *ACS nano* **12**, 4280-4285 (2018).
10. Wei X, *et al*. All-Weather Droplet-Based Triboelectric Nanogenerator for Wave Energy Harvesting. *ACS nano* **15**, 13200-13208 (2021).
11. Tang W, *et al*. Liquid-Metal Electrode for High-Performance Triboelectric Nanogenerator at an Instantaneous Energy Conversion Efficiency of 70.6%. *Advanced Functional Materials* **25**, 3718-3725 (2015).
