# Supplementary material for: Liquid-liquid triboelectric nanogenerator based on the immiscible interface of an aqueous two-phase system
Source: Nat Commun. 2022 Sep 9;13:5316. doi: 10.1038/s41467-022-33086-2 (PMC9463141; doi:10.1038/s41467-022-33086-2)
Supplement: Supplementary file 2 — Description of Additional Supplementary Files [file 41467_2022_33086_MOESM2_ESM.pdf]

Description of Additional Supplementary Files

File Name: Supplementary Movie 1
Description: Working demonstration of the L-L TENG.

File Name: Supplementary Movie 2
Description: Demonstration of the establishment of the aqueous two-phase system.

File Name: Supplementary Movie 3
Description: Demonstration of lightening the LED by the L-L TENG in series.

File Name: Supplementary Movie 4
Description: Demonstration of the L-L TENG as power supply for electronic devices after charging capacitor.

File Name: Supplementary Movie 5
Description: Demonstration of the biocompatibility of the L-L TENG as a suitable protein microreactor.
